# 3D MALDI Mass Spectrometry Imaging of a Single Cell: Spatial Mapping of Lipids in the Embryonic Development of Zebrafish

**DOI:** 10.1038/s41598-017-14949-x

**Published:** 2017-11-02

**Authors:** Maria Emilia Dueñas, Jeffrey J. Essner, Young Jin Lee

**Affiliations:** 1 Department of Chemistry, Iowa State University, Ames, IA 50011 USA; 2 Ames Laboratory-US DOE, Ames, IA 50011 USA; 3 Department of Genetics, Development and Cell Biology, Iowa State University, Ames, IA 50011 USA

## Abstract

The zebrafish (*Danio rerio*) has been widely used as a model vertebrate system to study lipid metabolism, the roles of lipids in diseases, and lipid dynamics in embryonic development. Here, we applied high-spatial resolution matrix-assisted laser desorption/ionization (MALDI)-mass spectrometry imaging (MSI) to map and visualize the three-dimensional spatial distribution of phospholipid classes, phosphatidylcholine (PC), phosphatidylethanolamines (PE), and phosphatidylinositol (PI), in newly fertilized individual zebrafish embryos. This is the first time MALDI-MSI has been applied for three dimensional chemical imaging of a single cell. PC molecular species are present inside the yolk in addition to the blastodisc, while PE and PI species are mostly absent in the yolk. Two-dimensional MSI was also studied for embryos at different cell stages (1-, 2-, 4-, 8-, and 16-cell stage) to investigate the localization changes of some lipids at various cell developmental stages. Four different normalization approaches were compared to find reliable relative quantification in 2D- and 3D- MALDI MSI data sets.

## Introduction

The zebrafish, *Danio rerio*, is a model vertebrate organism for studying and understanding developmental biology, drug discovery, and neurodegenerative diseases^1–3^. Zebrafish, a small tropical aquarium fish native to Southeast Asia, have a unique combination of genetic and experimental embryologic advantages that make them ideal for studying early development. Fertilized zebrafish embryos are accessible to observation and manipulation at all stages of development due to external fertilization and optical clarity^4^. Furthermore, zebrafish are readily available, inexpensive, hearty, easy to care for, and can lay hundreds of eggs at weekly intervals.

Zebrafish embryos have been used to study lipid metabolism, the roles of lipids in diseases, and lipid dynamics in embryonic development^5–7^. Recently, Fraher *et al*. conducted a lipidomic study using liquid chromatography-mass spectrometry and revealed that cholesterol, phosphatidylcholine (PCs), and triglycerides are the most abundant lipids in the zebrafish embryo. They demonstrated that lipids are processed within the yolk prior to mobilization to the embryonic body^5^. Desorption electrospray ionization mass spectrometry (DESI-MS) has also been used for direct MS analysis and imaging of lipids in individual zebrafish embryos across embryonic development (0, 24, 48, 72, and 96 hours post-fertilization)^7^. Metabolomics and lipidomics studies in zebrafish are of interest because these compounds have key biological functions, such as serving as energy storage sources, participating in cell signaling, and acting as essential components of cell membranes^8–10^. Exploring how metabolites and lipids are regulated is key to understanding biological pathways and developmental processes occurring in a biological system.

Traditional analytical approaches to study small metabolites and lipids require extensive sample preparation, laborious extractions, derivatizations, and previous knowledge of compounds of interest. Mass spectrometry imaging (MSI) has become a widely used analytical tool for these studies thanks to the recent development in sample preparation protocols and instrumentations^11–13^. MSI allows for two-dimensional visualization of the spatial distribution of biomolecules without extraction, purification, separation or labeling of analytes^14^. Moreover, many different classes of compounds, including unknowns, can be detected simultaneously from a single MSI experiment, which allows for direct cellular or sub-cellular mapping of biomolecules in high resolution and a high throughput manner.

Since biology occurs in organisms in three dimensions, it is not surprising that 3D imaging has had a noteworthy impact on many challenges in the life sciences^15^. Recently, imaging of intact biomolecules using mass spectrometry imaging has expanded to 3D analysis to determine volumetric molecular distribution within tissue specimens, agar plates, and 3D cell cultures^16^. The most common method of 3D imaging using mass spectrometry consists of collecting consecutive sections of a sample, analyzing each section individually using traditional 2-dimensional mass spectrometry imaging, and then stacking and reconstructing a final 3D imaging MS data set from the multiple 2-dimensional sets using computational methods^15^.

Our group has developed high-spatial resolution matrix-assisted laser desorption/ionization (MALDI)-MSI down to 5 μm resolution and utilized this for cellular or sub-cellular level imaging of plant metabolites^12,17,18^. Here, we present 3D MALDI-MSI of newly fertilized individual zebrafish (*Danio rerio*) embryos utilizing this high-spatial resolution. This is the first demonstration of 3D MSI for a single cell obtained with MALDI, revealing sub-cellular level localization of various lipid compounds. TOF-SIMS has been utilized for 3D MSI of single cells^19,20^, especially incorporating depth profiling as a way to achieve z-directional information; however, high mass compounds that can be analyzed by TOF-SIMS have been mostly limited to exogenous drug compounds due to significant fragmentations. In this analysis, we performed 3D MALDI-MSI on single zebrafish zygotes by acquiring MS imaging data set in positive and negative ion mode for alternative slides of 62 consecutive cross-sectional tissue sections. This allows for 3D visualization of more comprehensive lipid species from a single cell. Additionally, four different normalization approaches were compared to determine which of these can provide more representative results when comparing 2D MSI with the 3D volume reconstruction. Furthermore, full-scan MSI and MS/MS were acquired for embryos at different cell stages (1-, 2-, 4-, 8-, and 16-cell stage) to investigate the changes in phospholipid distribution during the early stages of zebrafish development.

## Results and Discussions

### Lipid profiles in three dimensions in one-cell stage zebrafish embryos

The newly fertilized zebrafish egg is in the zygote period until the first cleavage occurs^21^. In this period, the embryo is at the single-cell stage. Supplementary Figure S1a shows bright-field microscope images of serial cryo-sections of entire fertilized zebrafish embryo at the one-cell stage. Because of the nature and complexity of 3-dimensional imaging, sample preparation is a critical step in this study as it may be time-consuming and error-prone^22^. To diversify the chemical compounds that can be studied, both positive and negative ion mode data have been obtained for the same embryo. Odd number serial sections were analyzed in negative ion mode with DAN as a matrix, and even number serial sections were analyzed in positive ion mode with the binary matrix of DHB/Fe_3_O_4_ as a matrix. This will not only reduce the sample preparation time involved in the selection of the embryo, cryo-sectioning, and microscope inspection, but also minimize the sample-to-sample variation. Optical images (Supplementary Fig. S1b) of a zebrafish embryo at the one- and two-cell stages show the fertilized egg and uncleaved or cleaved blastodisc. The blastodisc and yolk can be readily distinguished in the microscope image, and their sizes are approximately 400 μm and 600 μm, respectively.

The overall workflow for MALDI-MSI of zebrafish embryos is shown in Supplementary Fig. S2. Zebrafish embryos were harvested at the one-cell stage, embedded in gelatin, and immediately flash frozen. Then, zebrafish embryos were cryo-sectioned at 10 μm thickness and collected through the entire embryo, from one side to the other, resulting in 62 sections. Optical images were then acquired prior to matrix application. MALDI-MS images were generated for phosphatidylcholine (PC), phosphatidylethanolamines (PE), and phosphatidylinositol (PI) molecular species in the serial sections using positive and negative mode data sets. For the 3D model reconstruction, all the MS images were computationally aligned to each other using the optical images as a guide, regaining the initial spatial relations prior to sectioning. False-colored images for each molecular species were adjusted to the same maxima for each species.

**Figure 1:**
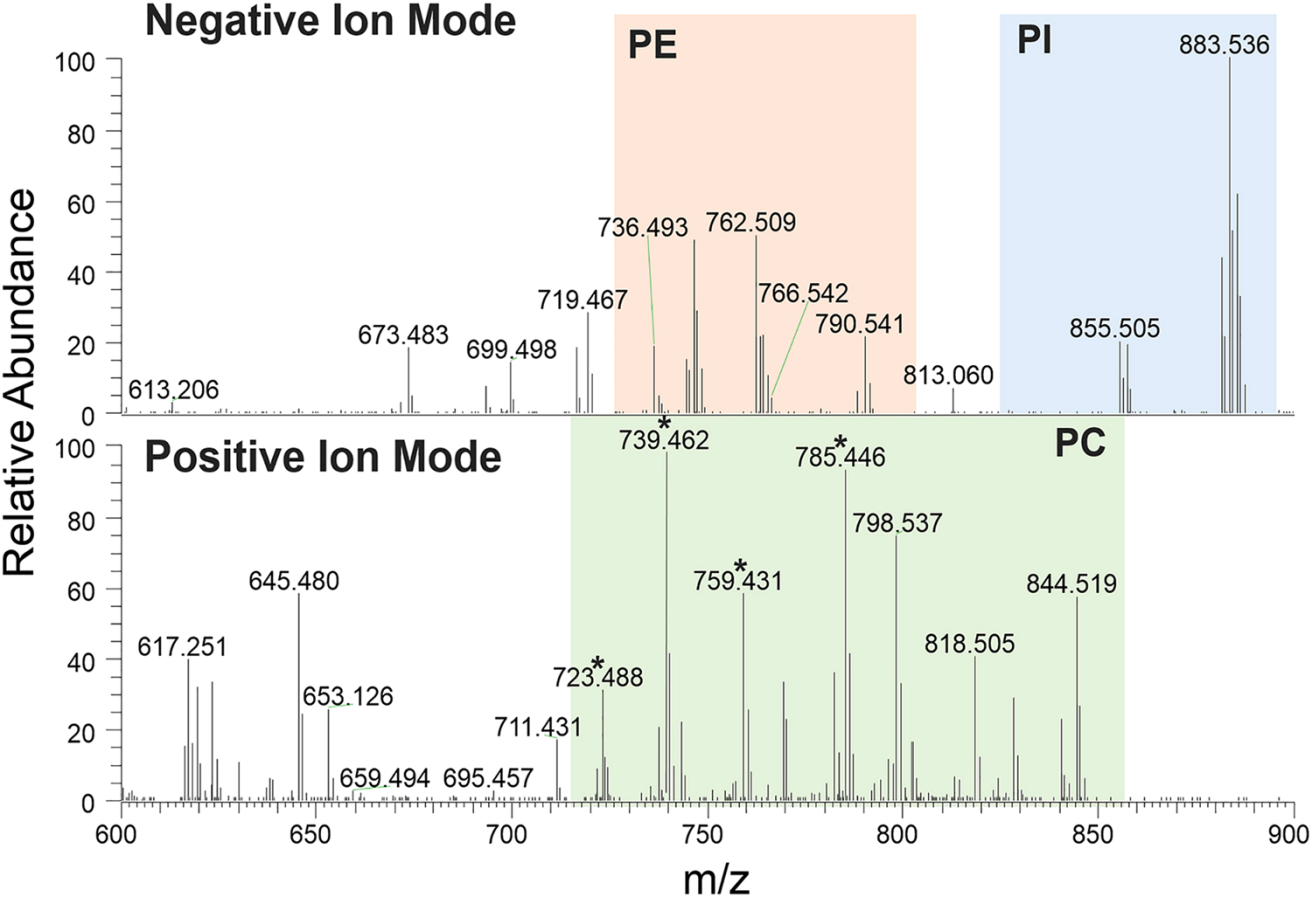
Representative spectra obtained in negative (top) and positive (bottom) ion mode. Spectral regions that are dominated by a particular phospholipid class are highlighted and labeled with the following class: PC, phosphatidylcholine; PE, phosphatidylethanolamine; and PI, phosphatidylinositol. *corresponds to trimethylamine loss (-N(CH_3_)_3_) from the headgroup of PCs.

**Figure 2:**
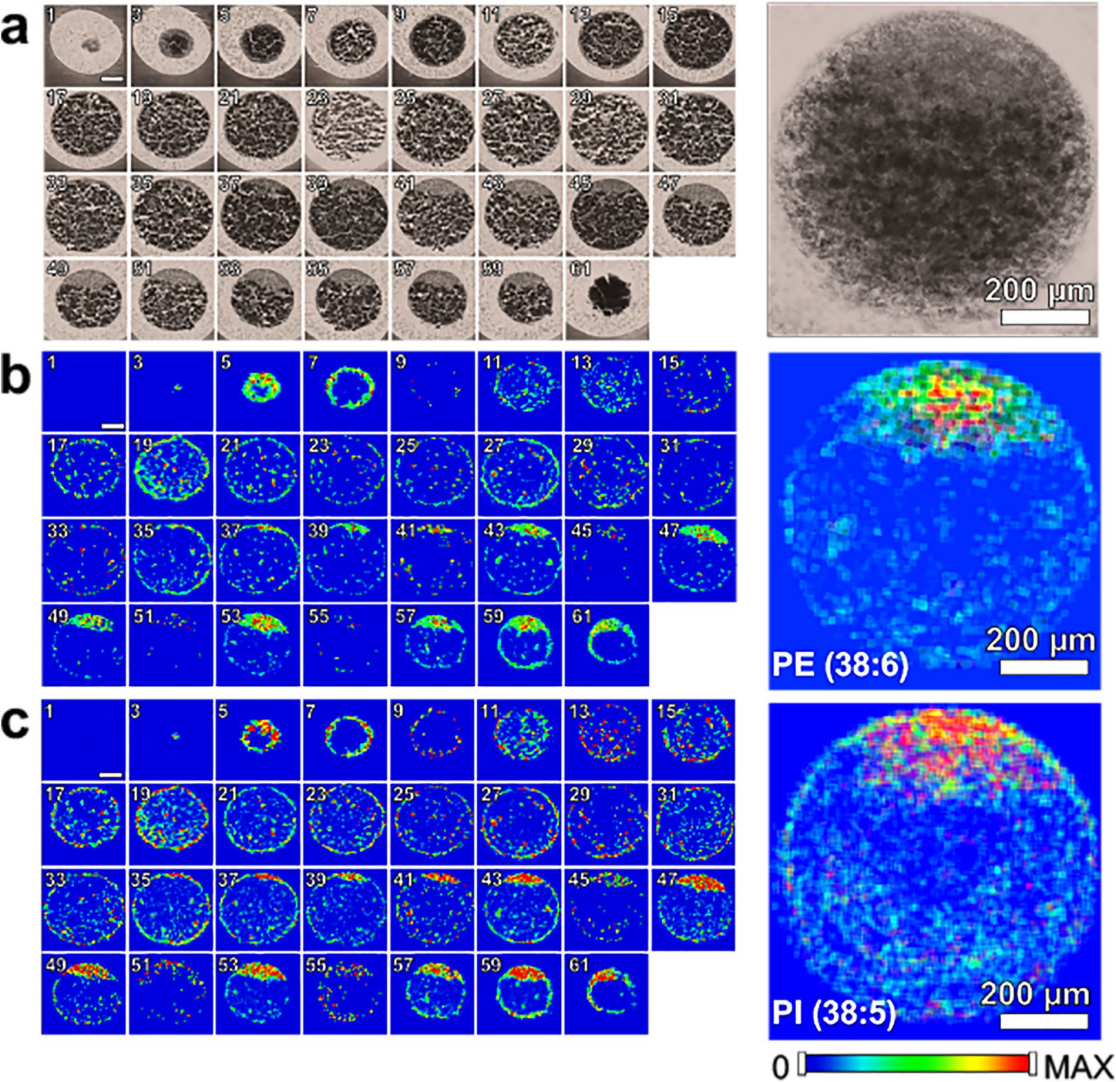
(**a**) Odd numbered optical images of fertilized zebrafish embryo at the one-cell stage. False color two-dimensional MALDI-MS images of (**b**) PE (22:6_16:0) at *m/z* 762.509 and (**c**) PI (18:0_20:5) at *m/z* 883.535. Projected images are shown on the right by overlaying all 2D images. All species were detected as deprotonated, [M-H]^−^.

**Figure 3:**
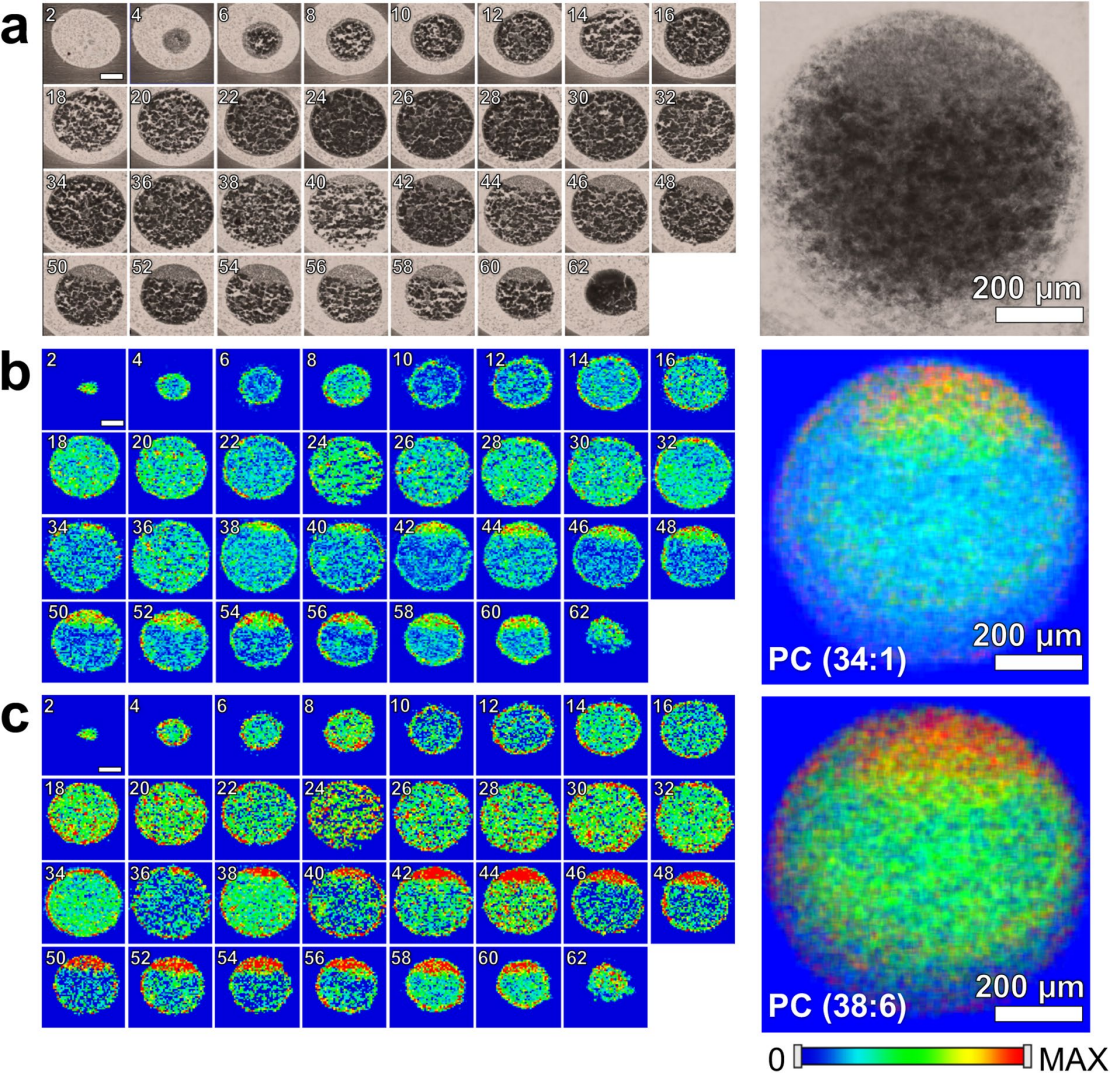
(**a**) Even numbered optical images of fertilized zebrafish embryo at the 1-cell stage. False color two-dimensional MALDI-MS images of (**b**) PC (18:1_16:0) at *m/z* 798.535 and (**c**) PC (16:0_22:6) at *m/z* 844.525. Projected images are shown on the right by overlaying all 2D images. All species were detected as potassiated, [M + K]^+^.

Representative single pixel mass spectra are shown in Fig. 1 for both ion modes. Spectral regions that are dominated by a particular phospholipid molecular species are highlighted and labeled for PC, PE, and PI. The spatial distribution of PE (22:6_16:0), PI (18:0_20:5), PC (18:1_16:0), and PC (16:0_22:6) were heterogeneous in localization in the different areas of the one-cell embryo (Figs 2 and 3). Overall, PE and PI are mostly absent or present minimally inside the yolk, while significant amounts of PCs are present, suggesting PCs are stored in the yolk. All three phospholipids are present in high abundance with symmetric distribution inside the blastodisc, as well as the boundary of yolk, which is not surprising considering their role as membrane lipids.

**Figure 4:**
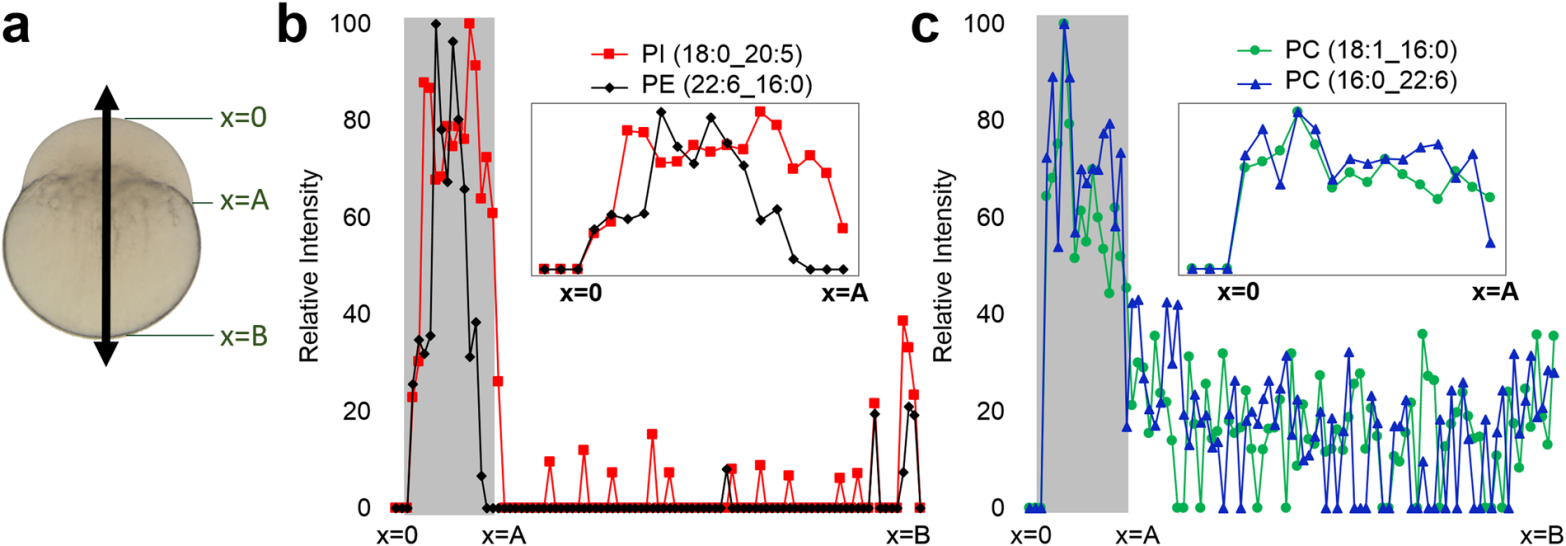
(**a**) Optical image of fertilized zebrafish embryo showing the arrow to indicate where the line profile is obtained. Line profile of ion intensities for (**b**) PI (18:0_20:5) and PE (22:6_16:0) and (**c**) PC (18:1_16:0) and PC (16:0_22:6), obtained from the tissue section 53 and 52, respectively.

To further illustrate these localizations, cross-section profiles were generated by mapping the signal intensities across the central part of zebrafish embryo as shown in Fig. 4, where the blastodisc region corresponds to x = 0~A, and the yolk region corresponds to x = A~B (Fig. 4a). PI (18:0_20:5), PC (18:1_16:0), and PC (16:0_22:6) show relatively homogenous distribution inside the blastodisc, whereas PE (22:6_16:0) shows elevated signal at the central region of the blastodisc (Fig. 4b–c). This slight change in distribution can also be visualized with a 2D-MSI overlay of PI (18:0_20:5), PE (22:6_16:0), and the optical image (Supplementary Fig. S3). Additionally, this approach indicates that PC species in the blastodisc is about four times higher than in the yolk. These distributions were consistent across multiple MALDI-MSI sections (Supplementary Fig. S4) as well as other major PE, PI, and PC species (not shown). Although sub-cellular level heterogeneous distribution of PE is clear from this analysis, we were not able to determine which sub-cellular organelles are responsible for this heterogeneity. It is mostly due to the fact that many sub-cellular organelles are being synthesized in a compact space, of which the distinction is beyond the spatial resolution of the current technology.

These species have also been detected in other metabolic studies on zebrafish embryos by GC-MS and LC-MS^3,23^ and via product ion scans using DESI-MS and nESI-MS^7^; however, detailed interpretation on the spatial distribution was limited due to their low spatial resolution of 250 µm. The PC and PE species are key markers for organogenesis^24^ while PI species are known to play a crucial role in intracellular signaling, RNA editing, protein phosphorylation, and gene transcription^7^, therefore their presence at the one-cell stage is not surprising. Assignments are based on accurate mass measurements, and their identities are also confirmed by separate MS/MS measurements for lipids (Supplementary Fig. S5 and Table S1). Additionally, in order to assign a degree of confidence to the assignments, molecular annotation was performed using the METASPACE^25^ annotation engine. Results are publically available at annotate.metaspace2020.eu and are shown in Table S2.

The video animations (Supplementary Videos S1–4) of the 3D distributions of PE (22:6_16:0), PI (18:0_20:5), PC (18:1_16:0), and PC (16:0_22:6) allow for rotation and visualization of the one-cell stage embryo and the yolk. These 3D reconstructions of lipid molecular species provide unprecedented volumetric chemical and spatial distribution of the lipidome in a single zebrafish embryo. Moreover, a 360° rotation visualization of PC (16:0_22:6) (Supplementary Fig. S6) reveals that the cross (e.g. 360°) and longitudinal visualization (e.g. 120°) are similar, verifying that the symmetry of the embryo is not affected when viewing that data in a 3D manner.

### Comparison of normalization strategies for relative quantification in three-dimension MSI of one-cell stage zebrafish embryo

Relative quantification is often achieved by normalizing analyte signals using a reference compound homogeneously present in the sample or summed ion signals from the same class of compounds^26^. This procedure minimizes variation of ion signals, and provides a better semi-quantitative representation. Improper normalization, however, may unintentionally distort the relative quantification^27^. Here, we compare four different normalization procedures to calculate mol% lipid composition from the 3D MALDI-MSI data set for one-cell stage zebrafish embryo. In our previous study on 3D MALDI-MSI analysis of Arabidopsis seeds^27^, we compared two different normalization methods, which can be expressed in equations (1) and (2).

1 $${{\hat{I}}}_{{P}_{i},volume}=\frac{{\sum }_{z}{\sum }_{x,y}{I}_{{P}_{i}}(x,y,z)}{{\sum }_{i}{\sum }_{z}{\sum }_{x,y}{I}_{{P}_{i}}(x,y,z)}$$

2 $${{\hat{I}}}_{{P}_{i},section}=\frac{1}{{\sum }_{z}{N}_{z}}\sum _{z}(\frac{{\sum }_{x,y}{I}_{{P}_{i}}(x,y,z)}{{\sum }_{x,y}{\sum }_{i}{I}_{{P}_{i(x,y,z)}}}){N}_{z}$$

Here, *I*_*Pi*_(x,y,z) is an ion signal of lipid species *P* with fatty acyl composition of *i* at a given tissue section of *z* and 2D position of *x* and *y*. Each *Î*_*Pi*_ represents a mol percentage of lipid species *P*_*i*_ within the same lipid class of *P*, normalized by different normalization methods. *N*_*z*_ corresponds to the number of voxels for the tissue section *z*.

Equation (1) represents a simple intensity average throughout the whole volume. In equation (1), the raw ion intensities for each lipid molecular species were summed together across all the tissue sections, and then normalized to the total ion signals from the same lipid class. This approach assumes that the signal at each voxel/section is a good representation of the molecular abundance in the whole embryo. This approach is easy to calculate but ignores any voxel-to-voxel or section-to-section analytical variation or the change of ionization efficiencies. Equation (2) represents 2D normalization performed at each section. In equation (2), the raw ion intensities for each lipid molecular species were summed together for each tissue section, then normalized by the same lipid class, and then averaged over the cross-sectional z-dimension. In this approach, it is assumed that section-to-section variation could be significant, thus normalization needs to be performed at each section. Because each section occupies a different volume (or different number of voxels, N_z_), two-dimensionally normalized mol percentage is weight-averaged across the section by the number of voxels in each section. This approach can minimize section-to-section variation (e.g., difference in matrix deposition) by normalizing at each section before weight-averaging over the entire sections, but it has a limitation because it ignores the quantitative differences between the voxels. By simply summing across a tissue section, the tissue or organelle-specific changes of ionization efficiencies are not taken into account. In our study on 3D analysis of Arabidopsis seeds, we could not find any difference between the two methods in the relative quantification of PC molecular species.

The third approach expressed in equation (3) is intended to minimize voxel-to-voxel analytical variation, by normalizing with the summed ion signals of the lipid class at each voxel, then averaging over the entire volume. This approach, however, does not take into account the quantitative difference between the voxels, and considers every voxel in equal weight. As a result, it may exaggerate the contribution from a voxel with low total lipid amount.

3 $${{\hat{I}}}_{{P}_{i},voxel}=\frac{1}{{\sum }_{z}{N}_{z}}\sum _{z}\sum _{x,y}\frac{{I}_{{P}_{i}}(x,y,z)}{{\sum }_{i}{I}_{{P}_{i}}(x,y,z)}$$

The forth normalization indicated in equation (4) is designed to minimize the above limitations, by normalizing with a matrix signal at each voxel before averaging over the entire volume. This approach assumes that matrix signal is a good representation of analytical variation.

4 $${{\hat{I}}}_{{P}_{i,m},voxel}=\frac{{\sum }_{z}{\sum }_{x,y}\frac{{I}_{{P}_{i}}(x,y,z)}{{I}_{m}(x,y,z)}}{{\sum }_{i}{\sum }_{z}{\sum }_{x,y}\frac{{I}_{{P}_{i}}(x,y,z)}{{I}_{m}(x,y,z)}}$$

**Figure 5:**
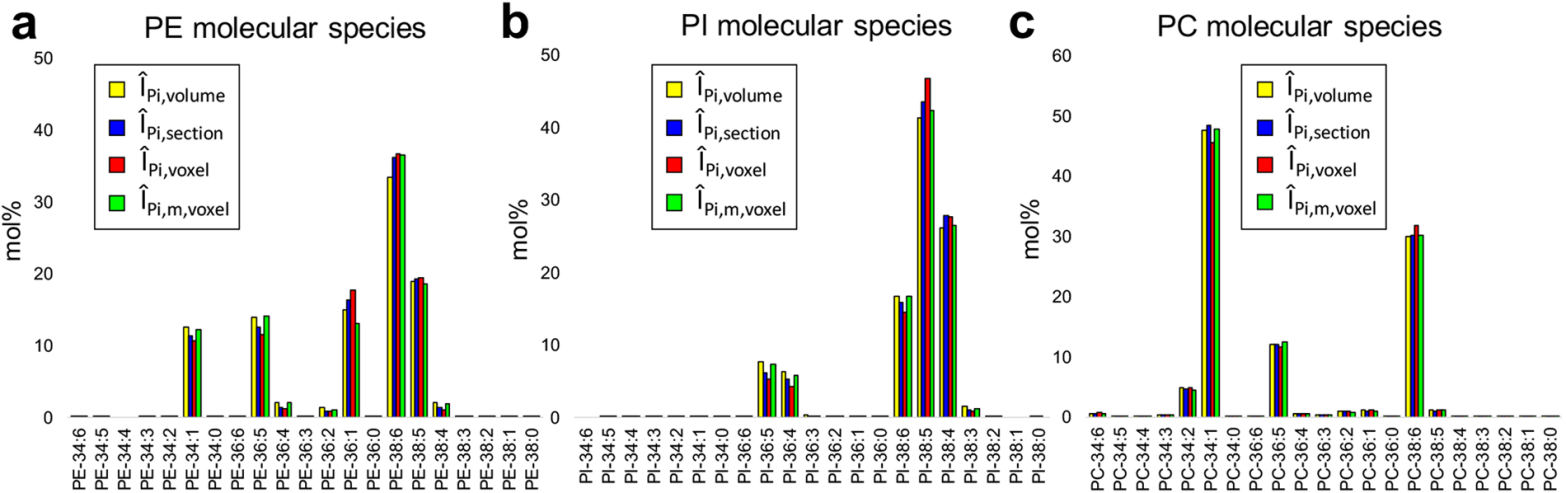
Comparison of the four normalization procedures in mol% calculation for the three lipid species of (**a**) PE, (**b**) PI, and (**c**) PC.

The four aforementioned normalization strategies were tested for relative quantification of three lipid classes (PC, PE, and PI) in the entire embryo as shown in Fig. 5.

It is known that the amount of salt or matrix can affect ionization efficiency^28^. The matrix application method adopted here provides a very homogeneous deposition, and there is a high potassium level in the DHB/Fe_3_O_4_ solution. As such, we do not expect any serious normalization artifacts, although the normalization process is expected to correct minor variations in ion signals. Only potassiated adducts are shown here, as they are the dominant species (88–95% of the total abundance of all adducts) and some protonated and sodiated adducts are indistinguishable (i.e. [PC-36:4 + H]^+^ and [PC-34:1 + Na]^+^ have *m/z* values of 782.569 and 782.568, respectively). Regardless, the same spatial distribution was obtained for all the adduct species present in positive ion mode (+H, +Na, and +K).

The three lipid classes show subtle but significant differences. The first and third approach are considered as two extremes, ignoring voxel-to-voxel variation of ionization efficiencies or over-emphasizing the variation of ionization efficiency at each voxel level, respectively. The last approach, normalization with a matrix peak at each voxel, seems to provide the best representation and typically gives the middle range values among the four methods.

2D MALDI-MSI data from a tissue section is often used to estimate lipid molar compositions, ignoring volumetric contribution^29^. We compared the lipid composition between 3D and 2D MALDI-MSI dataset, using the matrix ion signal at each voxel normalization, to see how good 2D data can represent 3D dataset. Considering the symmetrical shape of zebrafish embryos in single cell stage, a tissue section from the central area is expected to be a good representation of the whole volume. As shown in Supplementary Fig. S7, there is no dramatic difference between the 3D molecular compositions of PE species compared to those from two representative tissue sections. However, there is subtle but noticeable differences in their molecular compositions in some cases: i.e., PE-38:6 is 36% in 3D volume and 40% in section 47, and PC-34:1 is 48% in 3D and 52% and 45% in sections 46 and 52, respectively. This suggests that the 2D MALDI-MSI dataset from an optimal and representative tissue section could be a good semi-quantitative representation but may have up to a 10% difference compared to the 3D representation. It is important to note that these observations are valid for symmetric tissue samples like these, and may not be valid for other complex and heterogeneous samples. The mol% of these lipids obtained from 3D MALDI-MSI dataset is relatively in good agreement with those obtained from ESI-MS analysis of total extract (Supplementary Fig. S8). Minor difference between MALDI- and ESI-MS datasets are mostly attributed to the difference in electrospray ionization efficiencies between carbon chain length and unsaturation^30,31^, as well as imperfection in the normalization process used in semi-quantitative analysis in MALDI-MS.

### Lipid distribution of zebrafish embryos in early stages development

**Figure 6:**
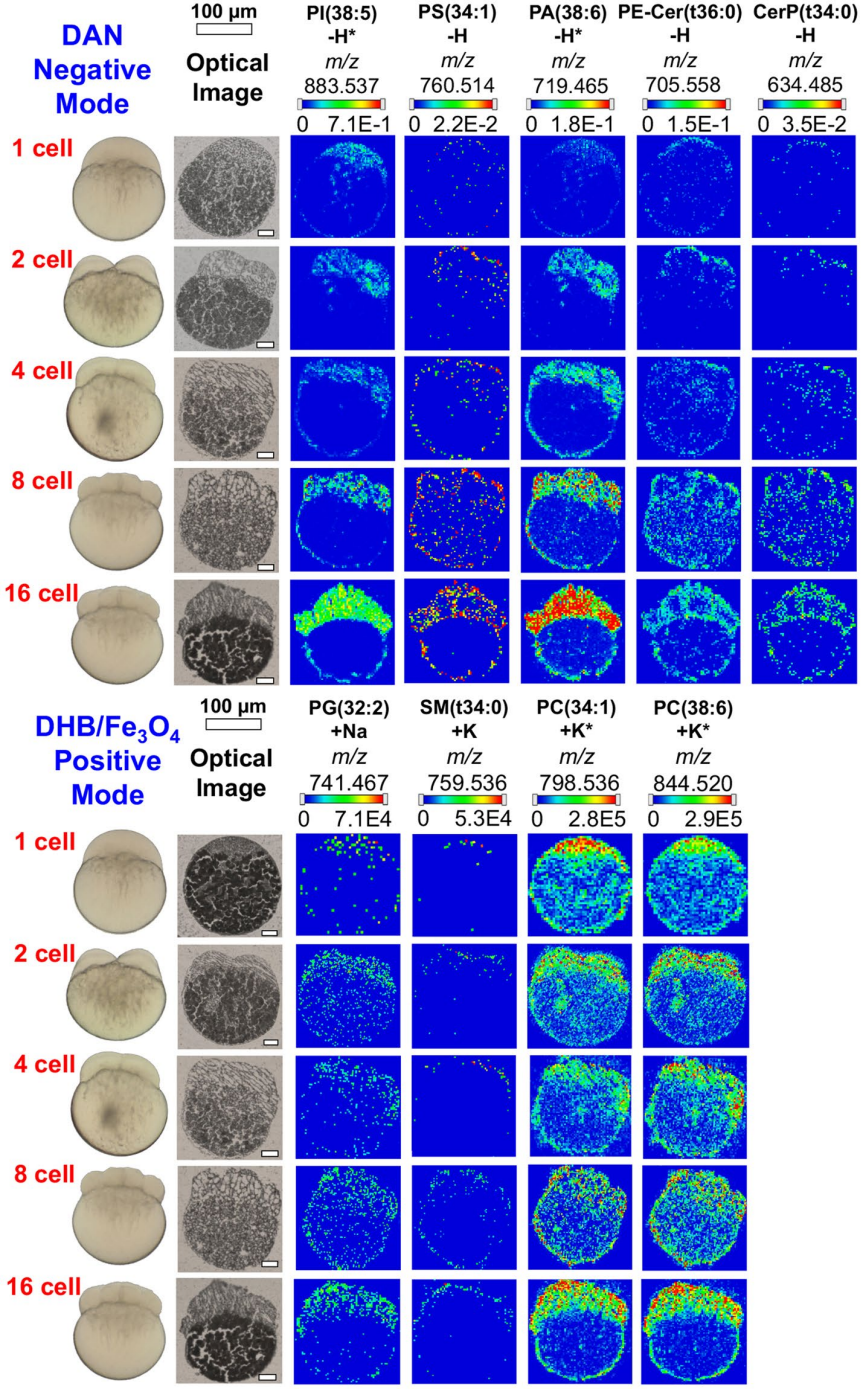
MALDI-MS images of selected lipid species in early developmental stages of zebrafish embryos. Peak assignments were based on accurate masses, except those marked by asterisk which were confirmed by MS/MS.

In an effort to better understand how the metabolites may change as the zebrafish embryo develops, a number of embryos at different stages (1-, 2-, 4-, 8-, and 16-cell stage) were evaluated using high-spatial resolution 2D MALDI-MS imaging (Fig. 6). These images were normalized with a matrix ion signal at each voxel (the forth normalization approach mentioned above). These images revealed spatially distinct areas of different lipid composition. Phosphatidic acids (PA) and PIs are present in the blastoderm region of the embryo while PCs are present in both the yolk and blastoderm. As development occurs, ceramide (Cer) containing lipids and phosphtatidylserines (PS) are localized in the cell boundaries [i.e. PS (34:1), PE-Cer(t36:0) and CerP(t34:0)], and sphingomyelins [SM(t34:0)] are present more on the outer membrane boundary of the embryo. The latter is consistent with the fact that sphingomyelin are an important component of the outer leaflet of mammalian cell membrane^32^. This is in contrast to other phospholipids (i.e. PI, PA, PG, and PC) that are present almost homogeneously inside the cells, suggesting they are essential components of not only the plasma cell membranes, but also membranes of various sub-cellular organelles. Lipid content in the yolk decreases at a later stage as they are being consumed for cell development^5^, which is especially clear for PCs at 16 cell stage. Due to low signal and poor quality of MS/MS spectra, these compounds have been assigned solely based on accurate mass but tentative identification has been supported by literature.

## Conclusions

The work described herein demonstrates, for the first time, that high-resolution MALDI-MSI can be applied for three dimensional chemical imaging of a single cell. We applied this platform to map the three-dimensional spatial distribution of phospholipid classes, phosphatidylcholine (PC), phosphatidylethanolamines (PE), and phosphatidylinositol (PI), for a newly fertilized individual zebrafish. All three phospholipid classes are present with symmetric distribution inside the blastodisc, as well as the boundary of the yolk, but each reveals different localization; PE shows heterogeneous sub-cellular localization highly abundant at the center of blastodisc. These 3D MALDI-MSI volumetric reconstructions were used to compare four normalization strategies and the normalization with the matrix ion signal at each voxel was found to provide the best representation for relative quantification. Using 2D MALDI-MSI, the distribution of phospholipids and ceramide containing lipids were observed in embryos at the 1-, 2-, 4-, 8-, and 16-cell stage revealing heterogeneous localization of different classes of lipids in the embryo. Future studies would reveal the details of embryo development with higher spatial resolution and visualization of small metabolites, as well as multi-modal imaging with fluorescence microscopy.

## Materials and Methods

### Chemicals

All the chemicals used in this study, 1,5-diaminonaphthalene (DAN, 97%), 2,5-dihydroxybenzoic acid (DHB, 98%), isopropanol (LC-MS grade) and gelatin from porcine skin (300 bloom), were purchased from Sigma-Aldrich (St. Louis, MO, USA). Iron oxide nanoparticles (NPs) were synthesized, as previously described^33^.

### Zebrafish embryo harvesting and sample preparation

All experimental protocols were approved by the Iowa State University Institutional Animal Care and Use Committee (Log # 11-06-6252-I) and are in compliance with American Veterinary Medical Association and the National Institutes of Health guidelines for the humane use of laboratory animals in research. The WIK wild type strain was obtained from the Zebrafish International Research Center (http://zebrafish.org/zirc/home/guide.php). Zebrafish were reared in an Aquatic Habitat system (Aquatic Ecosystems, Inc., Apopka, FL) and the fish were maintained on a 14-hr light/dark cycle at 27 °C. Newly fertilized embryos were obtained through natural mating of adult zebrafish and were collected and maintained at 28.5 °C in fish water (60.5 mg ocean salts/l). Embryos at the one-cell stage were transferred using a glass pipette into a Petri dish containing fish water, and embryo development was monitored under a stereomicroscope.

Upon reaching the desired cell stage (1-, 2-, 4-, 8-, and 16-cell stage), the zebrafish embryos were embedded in a 10% (w/v) gelatin solution and immediately frozen in liquid nitrogen, without dechorionation. The molds were transferred to a cryo-stat (Leica CM1850, Leica Microsystems; Buffalo Grove, IL, USA) set to a temperature of −20 °C and the samples were allowed to thermally equilibrate for 30 minutes. Zebrafish embryos were cryo-sectioned at 10 µm thickness. Sections were collected on Cryo-Jane tape (Leica Biosystems), and attached to pre-chilled glass slides. The prepared slides were placed on a chilled (−80 °C) aluminum block and lyophilized under moderate vacuum (~250 mtorr) for 1–1.5 hours. The lyophilizer was then slowly equilibrated to room temperature and atmospheric pressure, and sections were stored in a desiccator until matrix application for MALDI-MS imaging. All sections were imaged within 36 hours of sectioning.

### Mass spectrometry analysis

Binary matrix of a mixture of DHB and Fe_3_O_4_ NPs was applied by spraying with an oscillating capillary nebulizer and DAN was applied by sublimation, using the protocol previously reported^34,35^. DAN was applied to odd number serial sections and binary organic-inorganic matrix to even number serial sections. MS imaging data were collected using a MALDI-linear ion trap (LIT)-Orbitrap mass spectrometer (MALDI-LTQ-Orbitrap Discovery, Thermo Scientific; San Jose, CA, USA). The instrument was modified to use an external 355 nm frequency tripled Nd: YAG laser (UVFQ, Elforlight Ltd.; Daventry, UK). The laser energy used was 83–84% (~1 μJ/pulse) at a 60 Hz repetition rate. Laser optics is similar to previously described that allows down to 3–4 μm laser spot size with a 10X beam expander^17^. For this work, the laser spot size of ~7 μm and the raster size of 10 μm was used with a 5X beam expander. TunePlus and Xcalibur software (Thermo Scientific) were used to define imaging parameters and to acquire data, respectively. Mass spectra were acquired with 10 laser shots per spectrum in positive and negative mode using an Orbitrap mass analyzer (resolution of 30,000 at *m/z* 400) for *m/z* scan range of 100–1000. Raw mass spectral files acquired from the Orbitrap analyzer were used to generate images using ImageQuest software (Thermo Scientific; San Jose, CA, USA) with a mass window of ±0.003 Da and without normalization. Compound identification was based on accurate mass and confirmed by MS/MS analysis on replicate sections. MS/MS imaging was performed using the ion trap analyzer for selected ions using the same conditions as described for MS imaging. An isolation width of 2.0 Da and normalized collision energy of 35 were used. Cross-section profiles for MSI were generated by mapping the signal intensity across the zebrafish embryo using MSiReader (v. 0.09; North Carolina State University)^36^.

### Development of 3D models

Three-dimensional MALDI-MSI reconstructions was accomplished by collecting MALDI-MSI data sets for consecutive, serial sections of a sample, and reconstructing 3D images of each analyte from the multiple, stacked MALDI-MS images. First, 2D profile images of each specific *m/z* were produced using ImageQuest and saved in ‘.TIFF’ format; then, all the images were stacked together as a 3D model using TrakEM2 module^37–39^ of ImageJ (https://imagej.nih.gov/ij/; version 1.50e). Each 2D image was placed on top of the previous image, and adjusted for x, y position and rotation to properly orient the section relative to the previous sections. To ensure proper orientation and alignment, the 2D images were positioned using a half-transparent overlap with the previous image, and optical images obtained in parallel were used to guide the alignment. Once the 2D MALDI-MS images were stacked, aligned and transformed, Image J was used to visualize a 3D model and create 3D MALDI-MS image videos.

### Data availability statement

The datasets generated during and/or analyzed during the current study are available from the corresponding author on reasonable request.

## Electronic supplementary material

Supplementary Information
