# Supplementary material for: 3D MALDI Mass Spectrometry Imaging of a Single Cell: Spatial Mapping of Lipids in the Embryonic Development of Zebrafish
Source: Sci Rep. 2017 Nov 2;7:14946. doi: 10.1038/s41598-017-14949-x (PMC5668422; doi:10.1038/s41598-017-14949-x)
Supplement: Supplementary file 1 — Supplementary Information [file 41598_2017_14949_MOESM1_ESM.pdf]

Supporting Information for:

3D MALDI Mass Spectrometry Imaging of a Single Cell: Spatial Mapping of Lipids in the Embryonic Development of Zebrafish

Maria Emilia Dueñas[1], Jeffrey J. Essner[3] and Young Jin Lee[1,2*]

[1] Department of Chemistry, Iowa State University, Ames, IA, 50011, USA

[2] Ames Laboratory-US DOE, Ames, IA, 50011, USA

[3] Department of Genetics, Development and Cell Biology, Iowa State University, Ames, IA, 50011, USA

*Corresponding author: Dr. Young Jin Lee

0035A Roy J Carver Co-Lab

1111 WOI Road

Ames, IA 50011-3650

Tel: 515-294-1235

Email: yjlee@iastate.edu

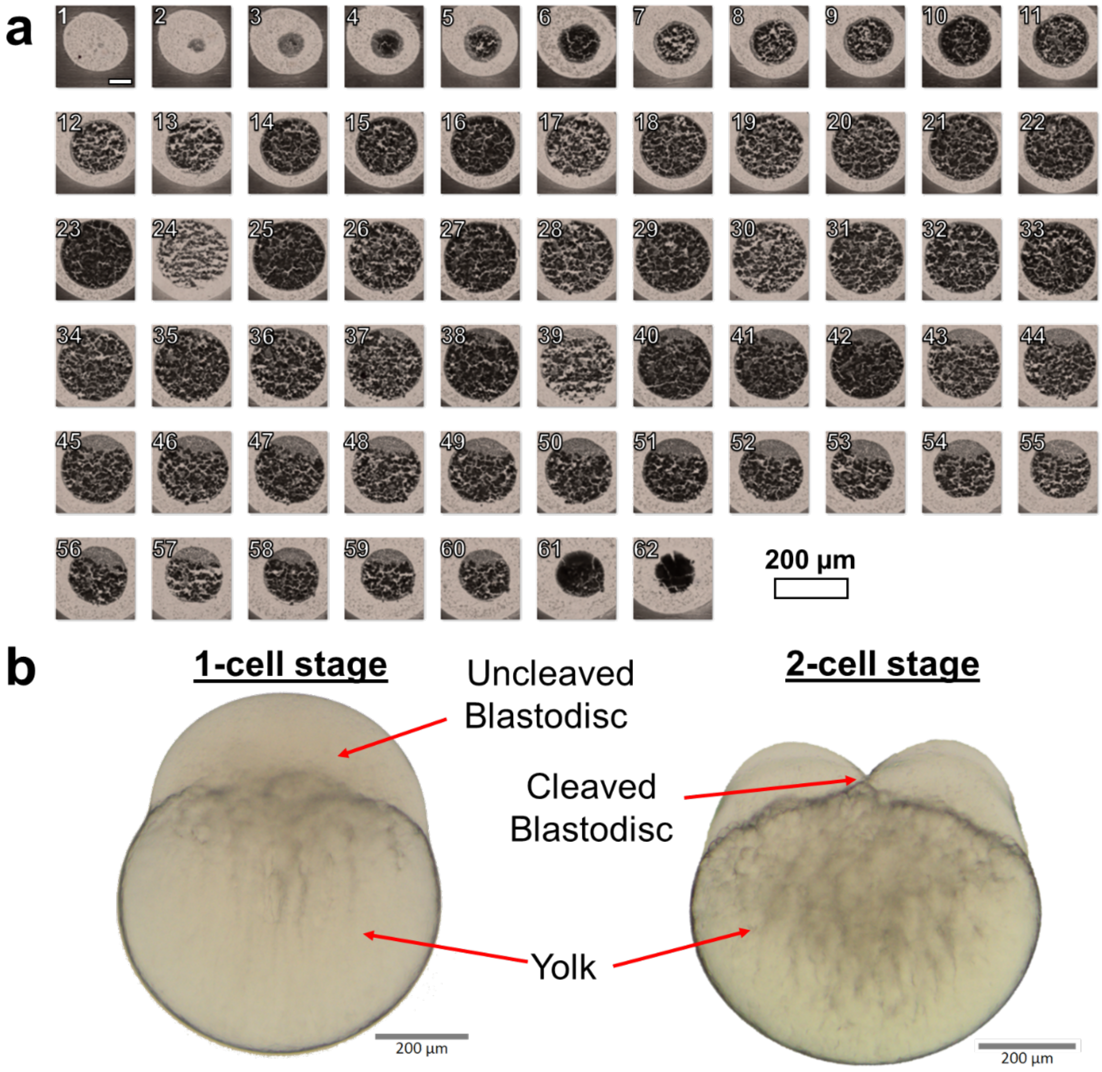

Fig. S1 (a) Bright-field images of serial cryo-sections of entire fertilized zebrafish embryo (*Danio rerio*) at the one-cell stage. The order of the sections is notated at the top left hand corner of each image. **(b)** Bright-field images of intact zebrafish embryos with the blastodisc, cleavage and yolk labeled.

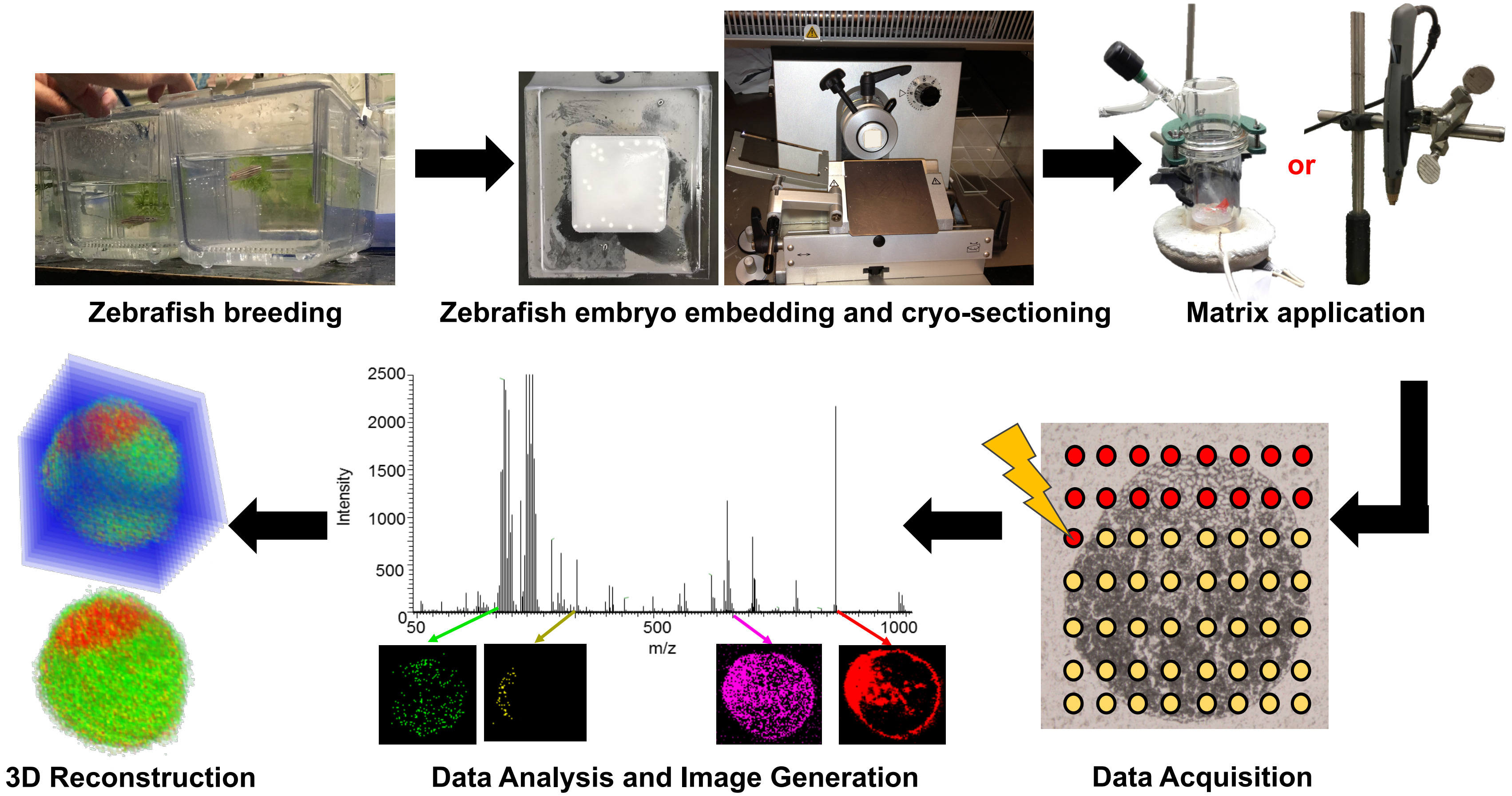

Fig. S2 Overall workflow for MALDI-MSI of zebrafish embryos

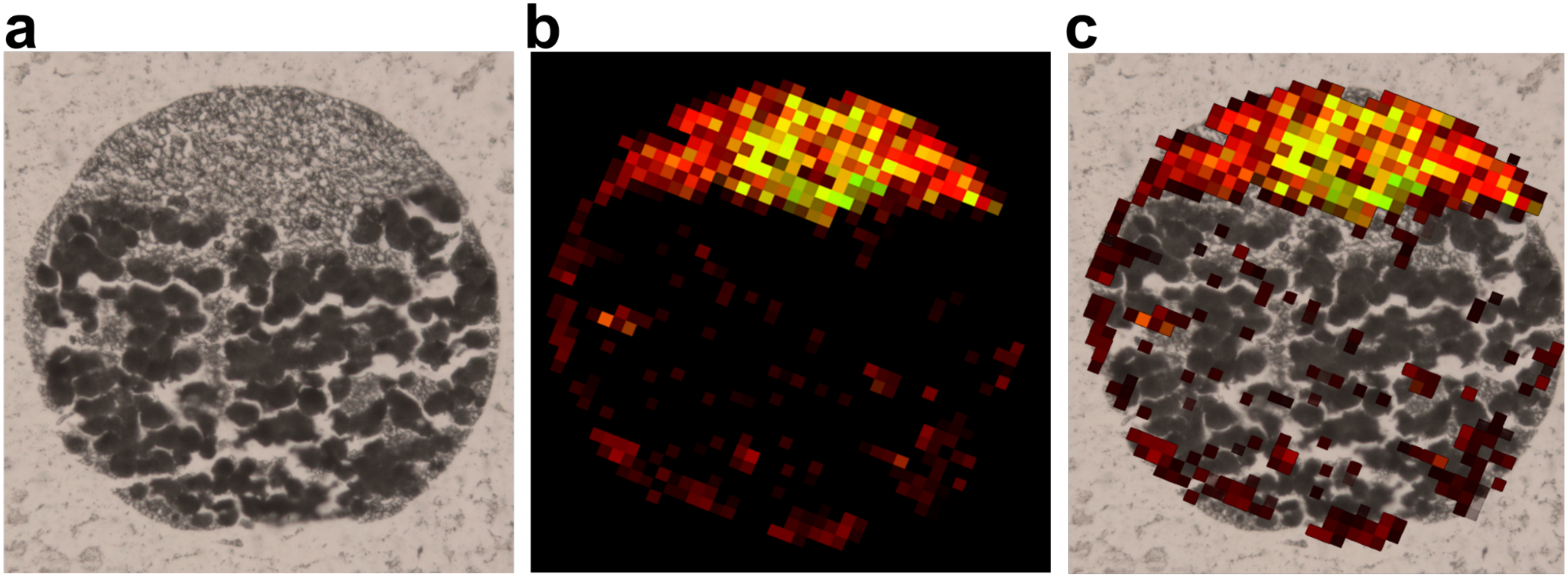

Fig. S3 (a) Optical image, **(b)** overlay MS image of PI (18:0_20:5) (red) and PE (22:6_16:0) (green), and **(c)** overlay of both lipids and the optical image obtained from tissue section 53.

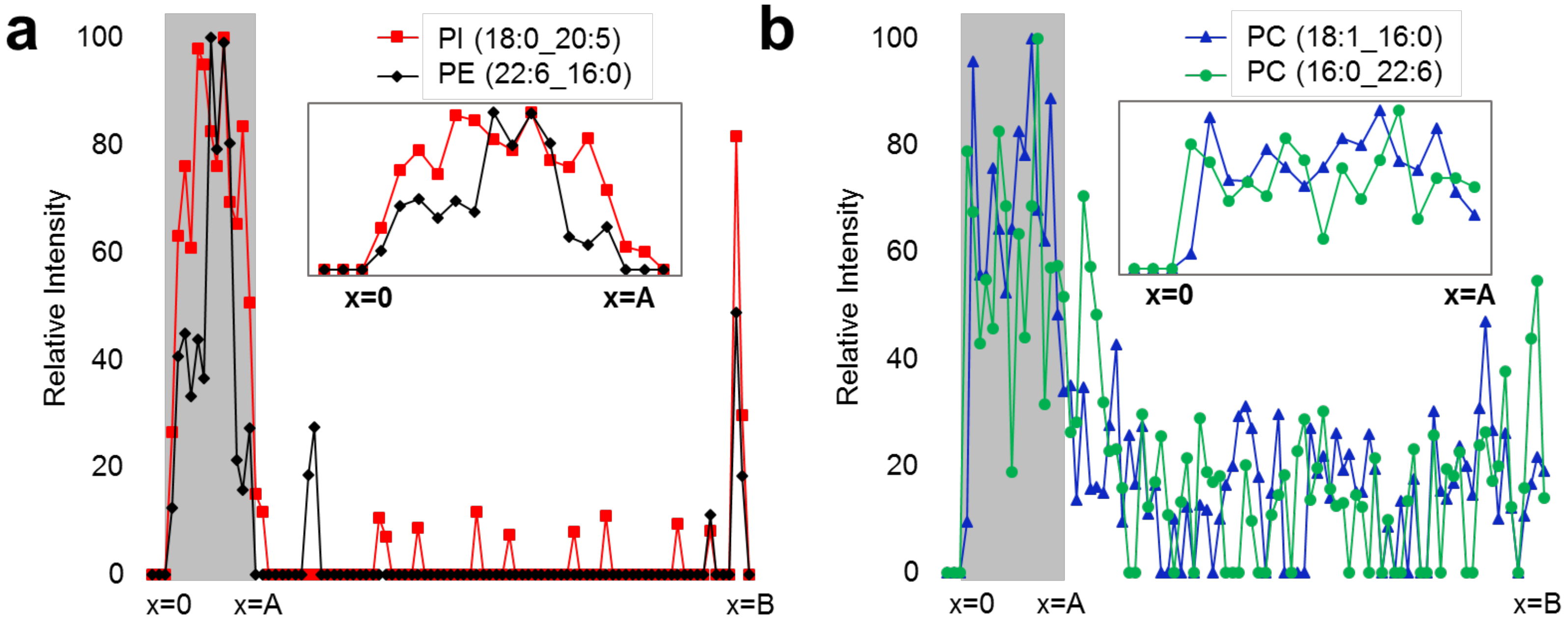

Fig. S4 Line profile of ion intensities for **(a)** PI (18:0_20:5) and PE (22:6_16:0) and **(b)** PC (18:1_16:0) and PC (16:0_22:6), obtained from the tissue section 47 and 46, respectively.

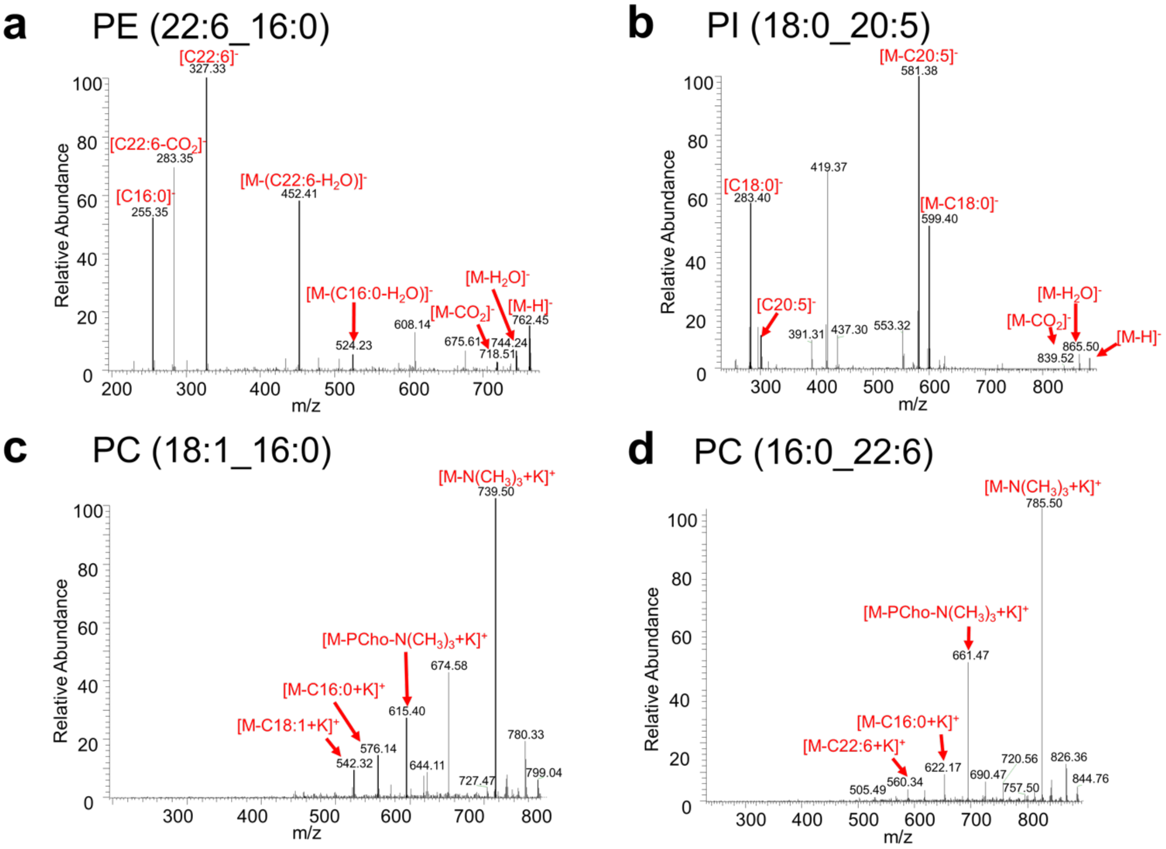

Fig. S5 MS/MS spectra of **(a)** PE (22:6_16:0), **(b)** PI (18:0_20:5), **(c)** PC (18:1_16:0), and **(d)** PC (16:0_22:6) obtained from zebrafish embryo at one-cell stage. PCho: phosphocholine head group. N$(CH_3)_3$: choline head group. PCho-N$(CH_3)_3$: Phosphocholine head group with trimethyl amine loss.

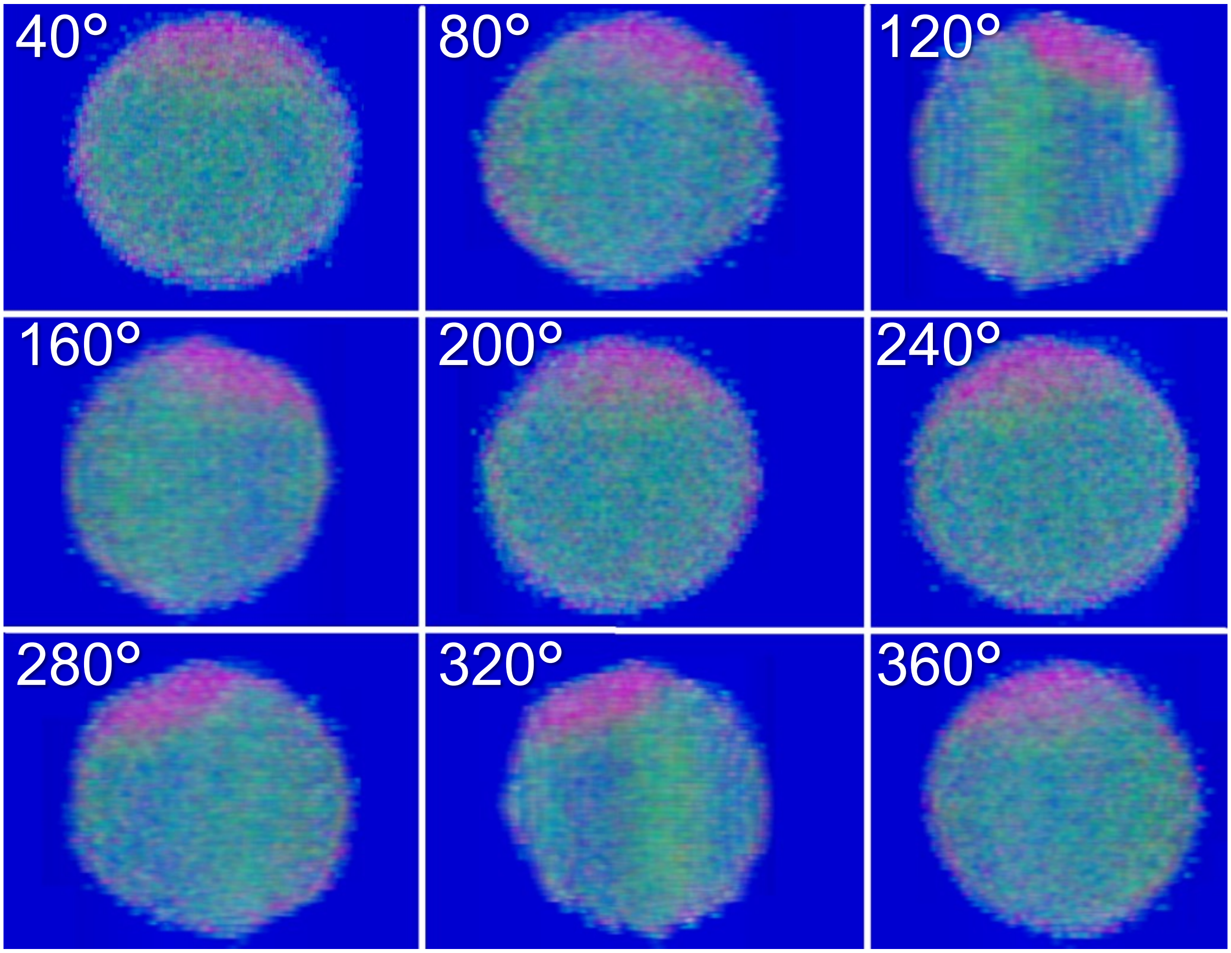

Fig. S6 2D visualization of PC (16:0_22:6) sliced along the various angles of 3D MS imaging data set.

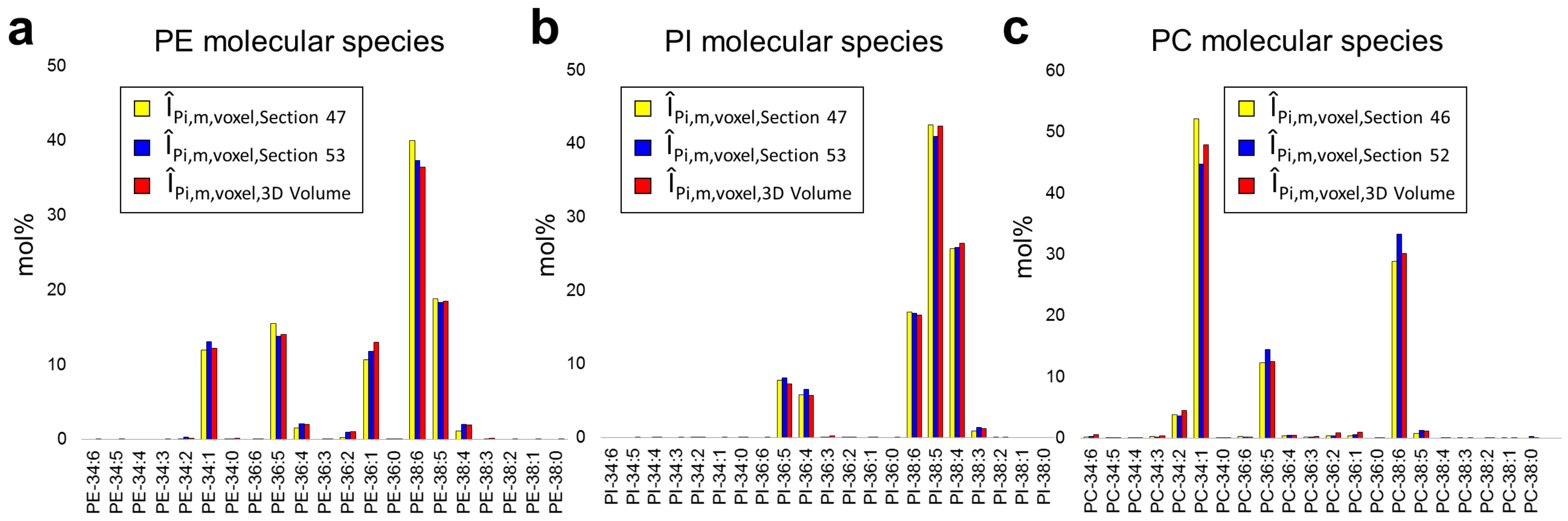

Fig. S7 Comparison of the mol% of **(a)** PE, **(b)** PI, and **(c)** PC molecular species obtained from 3D and 2D MALDI-MSI datasets, using the matrix ion signal for the normalization at each voxel.

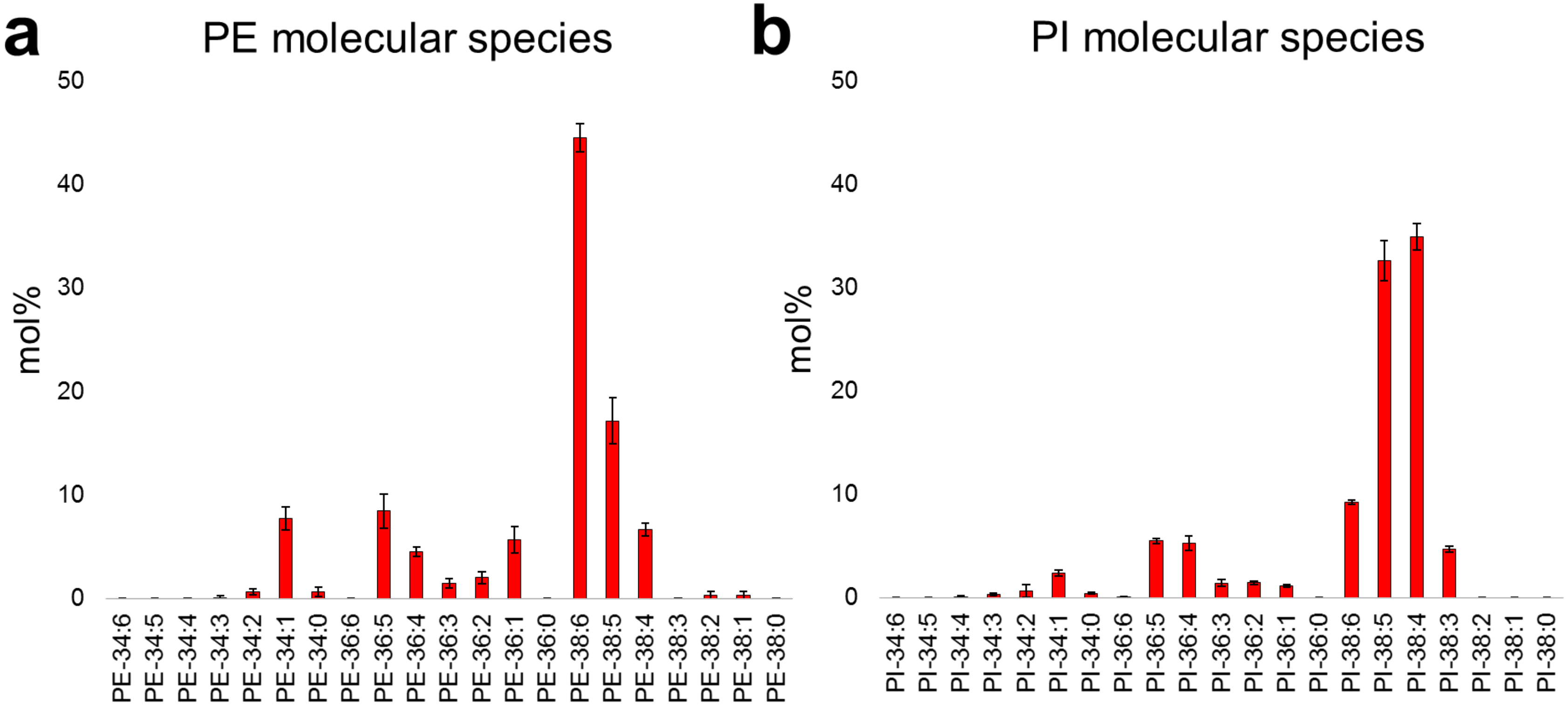

Fig. S8 Mol% of **(a)** PE and **(b)** PI molecular species obtained by ESI-MS of total extract of one-cell stage zebrafish embryos.

Table. S1 Tentatively identified compounds

ID[a]	m/z	Species Observed	Error (ppm)	Supporting MS/MS ions[b]
CerP(t34:0)	634.485	-H	-4.7	-
PA-38:6	719.465	-H	1.4	463 (M-16:0), 327 (22:6-H), 255 (16:0-H)
PC-34:1	798.536	+K	5.0	739 (M-$(CH_3)_3$+K), 615 (M-PCho-N$(CH_3)_3$+K), 576 (M-16:0+K), 542 (M-18:1+K)
PC-34:2	796.522	+K	3.8	737 (M-N$(CH_3)_3$+K), 613 (M-PCho-N$(CH_3)_3$+K)
PC-36:1	826.567	+K	6.0	767 (M-N$(CH_3)_3$+K), 643 (M-PCho-N$(CH_3)_3$+K)
PC-36:2	824.553	+K	3.6	765 (M-N$(CH_3)_3$+K), 641 (M-PCho-N$(CH_3)_3$+K)
PC-36:3	822.536	+K	4.9	763 (M-N$(CH_3)_3$+K), 639 (M-PCho-N$(CH_3)_3$+K)
PC-36:4	820.521	+K	4.9	761 (M-N$(CH_3)_3$+K), 637 (M-PCho-N$(CH_3)_3$+K)
PC-36:5	818.506	+K	3.7	759 (M-N$(CH_3)_3$+K), 635 (M-PCho-N$(CH_3)_3$+K), 562 (M-18:1+K)
PC-36:6	816.491	+K	3.7	757 (M-N$(CH_3)_3$+K), 633 (M-PCho-N$(CH_3)_3$+K)
PC-38:5	846.536	+K	5.9	787 (M-$(CH_3)_3$+K), 663 (M-PCho-N$(CH_3)_3$+K)
PC-38:6	844.521	+K	4.7	785 (M-$(CH_3)_3$+K), 661 (M-PCho-N$(CH_3)_3$+K), 622 (M-16:0+K), 560 (M-22:6+K)
PE-34:1	716.523	-H	1.4	281 (18:1-H) 255 (16:0-H)
PE-34:2	714.507	-H	1.4	-
PE-36:1	744.555	-H	-1.3	506 (M-16:0-H_2O), 480 (M-18:1-H_2O), 281 (18:1-H), 255 (16:0-H)
PE-36:2	742.540	-H	-1.3	480 (M-18:2-H_2O), 279 (18:2-H)
PE-36:4	738.510	-H	-2.7	476 (M-18:2-H_2O), 279 (18:2-H)
PE-36:5	736.494	-H	-2.7	452 (M-20:5-H_2O), 301 (20:5-H), 255 (16:0-H)
PE-38:3	768.555	-H	-1.3	-
PE-38:4	766.540	-H	-1.3	-
PE-38:5	764.525	-H	-1.3	279 (18:2-H), 277 (18:3-H)

PE-38:6	762.509	-H	-1.3	524 (M-16:0-H_2O), 452 (M-22:6-H_2O), 327 (22:6-H), 255 (16:0-H)
PE-Cer(t36:0)	705.558	-H	-2.8	-
PG-32:2	741.468	+Na	0.0	-
PI-36:4	857.520	-H	-2.3	577 (M-18:2), 279 (18:2-H)
PI-36:5	855.504	-H	-1.2	599 (M-16:0), 553 (M-20:5), 301 (20:5-H). 255 (16:0-H)
PI-38:3	887.567	-H	-1.1	-
PI-38:4	885.552	-H	-2.3	-
PI-38:5	883.535	-H	-1.1	599 (M-18:0), 581 (M-20:5), 301 (20:5-H), 283 (18:0-H)
PI-38:6	881.521	-H	-2.3	301 (20:5-H), 281 (18:1-H)
PS-34:1	760.514	-H	-1.3	-
SM-t34:0	759.536	+K	6.6	-

a. Assignments were based on accurate mass search on Metlin and manual MS/MS interpretation.
b. Fragment assignments are shown in parenthesis. N(CH3)3: choline head group, PCho-N(CH3)3: phosphocholine head group with trimethyl amine loss. 16:0, 18:0, 18:1, 18:2, 18:3, 20:5, 22:6: C16:0, C18:0, C18:1, C18:2, C18:3, C20:5, C22:6 fatty acid, respectively.

Table. S2 Metaspace Annotation

		METASPACE ANNOTATION				
**ID**	**m/z**	**MSM**	**FDR**	**rhoSpatial**	**rhoSpectral**	**rhoChaos**
CerP(t34:0)	634.485					
PA-38:6	719.465					
PC-34:1	798.536	0.686	0.050	0.736	0.937	0.996
PC-34:2	796.522					
PC-36:1	826.567					
PC-36:2	824.553					
PC-36:3	822.536					
PC-36:4	820.521					
PC-36:5	818.506					
PC-36:6	816.491					
PC-38:5	846.536					
PC-38:6	844.521	0.666	0.050	0.718	0.930	0.997
PE-34:1	716.523	0.377	0.050	0.428	0.891	0.989
PE-34:2	714.507					
PE-36:1	744.555	0.210	0.050	0.248	0.869	0.977
PE-36:2	742.54					
PE-36:4	738.51	0.084	0.100	0.102	0.853	0.968
PE-36:5	736.494	0.377	0.050	0.421	0.903	0.991
PE-38:3	768.555					
PE-38:4	766.54					
PE-38:5	764.525	0.327	0.050	0.372	0.889	0.989
PE-38:6	762.509	0.597	0.050	0.647	0.928	0.995
PE-Cer(t36:0)	705.558					
PG-32:2	741.468					
PI-36:4	857.52	0.229	0.050	0.267	0.869	0.986
PI-36:5	855.504					
PI-38:3	887.567					
PI-38:4	885.552	0.565	0.050	0.628	0.908	0.992
PI-38:5	883.535	0.577	0.050	0.634	0.915	0.994
PI-38:6	881.521					
PS-34:1	760.514					
SM-t34:0	759.536					
